# The influence of ecological and geographic limits on the evolution of species distributions and diversity

**DOI:** 10.1111/evo.13563

**Published:** 2018-08-07

**Authors:** Leonel Herrera‐Alsina, Alex L. Pigot, Hanno Hildenbrandt, Rampal S. Etienne

**Affiliations:** ^1^ Groningen Institute for Evolutionary Life Sciences University of Groningen Groningen 9700 CC The Netherlands; ^2^ Centre for Biodiversity and Environment Research, Department of Genetics, Evolution and Environment University College London London WC1E 6BT United Kingdom

**Keywords:** Diversity‐dependence, geographic area, geographic range size, local carrying capacity, species diversification, species saturation

## Abstract

The role of ecological limits in regulating the distribution and diversification of species remains controversial. Although such limits must ultimately arise from constraints on local species coexistence, this spatial context is missing from most macroevolutionary models. Here, we develop a stochastic, spatially explicit model of species diversification to explore the phylogenetic and biogeographic patterns expected when local diversity is bounded. We show how local ecological limits, by regulating opportunities for range expansion and thus rates of speciation and extinction, lead to temporal slowdowns in diversification and predictable differences in equilibrium diversity between regions. However, our models also show that even when regions have identical diversity limits, the dynamics of diversification and total number of species supported at equilibrium can vary dramatically depending on the relative size of geographic and local ecological niche space. Our model predicts that small regions with higher local ecological limits support a higher standing diversity and more balanced phylogenetic trees than large geographic areas with more stringent constraints on local coexistence. Our findings highlight how considering the spatial context of diversification can provide new insights into the role of ecological limits in driving variation in biodiversity across space, time, and clades.

Two coexisting insect species in a pond in Palermo made G. E. Hutchinson wonder about the limited size of this community in stark contrast to the huge number of species on Earth (Hutchinson 1959). Ever since then, the notion that there are ecological and geographical limits to the diversity of species found on Earth has been a central tenet of ecology and evolution. For instance, increases in diversity over geological time have often been linked to the accessing of novel regions of geographic or ecological niche space (Benton 2009), while differences in richness between clades and regions are typically associated with differences in environmental conditions or geographic area that are thought to limit the total number of species that can be packed within a region (Rabosky 2009; Ezard et al. 2011). However, it has also been argued that such limits–if they even exist–do not impose an important constraint on diversity, which instead may be largely controlled by historical factors (Wiens 2011). According to this argument, variation in diversity primarily involves nonequilibrium explanations, including differences in the time available for diversification, rates of colonization, speciation, or extinction (Ricklefs and Bermingham 2001; Wiens and Donoghue 2004; Jetz and Fine 2012). Despite decades of interest, opinions regarding the relative importance of limits to diversity in driving macro‐ecological and evolutionary patterns remain divided (Harmon and Harrison 2015; Rabosky and Hurlbert 2015).

Resolving the debate about whether species richness is bounded is challenging because present‐day patterns of diversity are the result of processes acting over a variety of spatial and temporal scales, from the local ecological dynamics within assemblages, to the processes of geographic range expansions and speciation operating over thousands to millions of years. Ultimately, any limit to the total number of species that can be packed within a region must arise through two primary routes. First, at a local level, species diversity will be limited by the size of available ecological niche space which places an upper bound on the number of species that can coexist within an assemblage (Macarthur 1965; Brown et al. 2001). Second, species occupying identical ecological niches may coexist at a regional scale, if they occur in different geographic places (i.e., different assemblages) (Levins and Culver 1971; Atkinson and Shorrocks 1981; Ruokolainen and Hanski 2016; Mehrparvar et al. 2017). Thus at the level of entire regions, richness will be a function of both the number of species that can be packed within a given assemblage (i.e., ecological niche space) and the number of assemblages available for colonization (i.e., geographic space).

Tests of whether diversity is bounded typically rely on comparative approaches, examining how diversity varies across different regions, clades, and over evolutionary time (Cornell and Lawton 1992; Cornell 2013; Pinto‐Sánchez et al. 2014). A key prediction of bounded models of species diversity is that the rate of species diversification should slow down over time as richness approaches an ecological or geographic limit (Nee et al. 1994; Phillimore and Price 2008; Etienne et al. 2012; Price et al. 2014). In contrast, if diversity were unbounded, then richness is expected to fluctuate randomly or increase exponentially over time (Alroy et al. 2008). Many studies have attempted to test these predictions by inferring the temporal dynamics of diversification from the fossil record and from reconstructed phylogenies of extant species (Stanley 1973; Alroy 2010; Ezard et al. 2011). However, these patterns of species diversification may be difficult to interpret for at least two key reasons. First, it has been argued that even if local communities are saturated with species, regional diversity may still increase over time if there are continued geographic opportunities for speciation and this outpaces rates of regional extinction (Cornell 2013). Second, even if rates of diversification slow down over time this need not necessarily imply that local coexistence is limited by niche availability, because this same pattern can also arise through other mechanisms (Moen and Morlon 2014), including methodological artifacts (model misspecification, Revell et al. 2005; or incomplete sampling, Nee et al. 1994; Pybus and Harvey 2000), temporal lags in the completion of speciation (Etienne and Rosindell 2012) or neutral geographic range dynamics (Pigot et al. 2010). Patterns of species diversification alone may therefore not provide enough information to discriminate bounded versus unbounded models of diversity (Cornell 2013).

A related problem is that most models of species diversification assume a direct link between the total species diversity of a clade or region and the fundamental rates of speciation and extinction. In reality, however, these rates are likely to respond to regional richness indirectly through a chain of intermediate stages in which geographical factors play a central role (Price 2008). In particular, widespread species are more likely to give birth to new species whereas small‐ranged species are more likely to go extinct by chance (Gaston 1998). As a result, sustained diversification requires that newly formed species are able to expand their geographic ranges, thus avoiding extinction and providing new opportunities for speciation. However, range expansion requires species to colonize areas that often already contain other species (Moreno et al. 2006; Ricklefs 2012). As the number of species in local communities increases, resource and niche availability are expected to decline until further colonization is prevented or at least strongly inhibited (Price et al. 2014). By constraining the opportunities for geographic range expansion, local limits to diversity may therefore reduce rates of speciation and increase rates of extinction, thus regulating overall regional diversity (Rosenzweig 1975; Pigot et al. 2010). This chain of causation is generally overlooked by current models of diversification, where diversity‐dependence is assumed to act globally and where it is the richness of the entire clade that influences the speciation or extinction rate of each individual lineage even though many of these lineages will not be interacting (Xu and Etienne 2018). Understanding the macroevolutionary and macroecological patterns expected in the presence or absence of limits to diversity therefore requires developing models that more explicitly account for the key ecological and geographic mechanisms linking variation in standing‐diversity with rates of speciation and extinction over time.

Here, we study how local ecological limits to coexistence and regional geographic constraints influence the dynamics of species diversification and geographic range evolution using a stochastic, spatially explicit simulation model of speciation, colonization, and local extinction. We model the spatial dynamics of diversification on a two‐dimensional gridded domain where each cell represents a local assemblage and where the area of the region (*A*) sets an upper bound on the number of populations or geographic range size of each species. We model the effects of ecological limits to coexistence, by assuming that only a limited number of species (*K*_L_) can be packed within any given local assemblage, so that once saturated no more species can colonize the assemblage until a resident species has become locally extinct. Thus, we model ecological limits to the number of coexisting species rather than limits to the number of individuals (e.g., Ranjard et al. 2018; Hurlbert and Stegen 2014a, b). We assume that species are ecologically neutral in the sense that their constituent populations are governed by identical rates of extinction, speciation, and colonization (Economo and Keitt 2008; Etienne and Rosindell 2011). In this model, the potential total diversity of the region (*K*_R_) is determined by the number of assemblages in the region *A* and the local ecological limit *K*_L_ of each assemblage, which we assume is uniform across space.

We first describe the general dynamics of this model, focusing on the links between the saturation of local assemblages, the dynamics of species geographic ranges and the diversification of species at the regional scale. By examining how these dynamics vary according to different parameter combinations we address the following key questions. First, how do local ecological limits to diversity *K*_L_ and constraints on regional area *A* influence both the true and reconstructed temporal dynamics of species diversification, and how are these effects modulated by relative rates of speciation, extinction, and colonization? Second, do regional area *A* and local ecological limits *K*_L_ have equivalent effects on the dynamics of species diversification or do they limit diversity in fundamentally different ways? Finally, how do ecological and geographic limits, influence other key dimensions of biodiversity, including species geographic range size, the relationship between range size and species evolutionary age and the variation in species richness across clades (i.e., phylogenetic tree balance)?

## Methods

### MODELING THE SPATIAL DYNAMICS OF DIVERSITY‐DEPENDENT DIVERSIFICATION

We constructed a continuous‐time stochastic Markov model to describe the dynamics of speciation, extinction, and colonization on a square gridded domain with hard boundaries. The grid contains *A* cells and each cell can contain up to *K*_L_ different species. By independently varying *A* and *K*_L_ we examine the effects of varying both the total diversity limit of the entire region *K*_R_ and also the degree to which this potential diversity is partitioned mainly across (i.e., *A* > *K*_L_) or mainly within (i.e., *A* < *K*_L_) local assemblages. The simulation starts with a single species occupying a single cell, randomly chosen from within the domain. Species expansion is modeled by selecting a population (i.e., a single cell from within a species’ range) with probability rate γ, and then randomly selecting one of the four adjacent (in the cardinal directions) cells for colonization. If the species is already locally present in this target cell, then colonization has no effect. Furthermore, colonization is prevented if the target cell is already saturated with species (i.e., local richness equals *K*_L_). In this way, local ecological limits act by preventing the geographic expansion of species ranges (Price 2008). The extinction of populations (hereafter called local extinction) occurs with a per‐population probability μ and is modeled by removing a randomly selected population. The stochastic processes of colonization and local extinction give rise to changes in both local diversity and the geographic range size of species over time. Species extinction takes place when the last population of a species becomes extinct. Speciation occurs at a per‐capita rate λ and is modeled by randomly selecting a single population from within the range of the species and labeling this as a new species. Thus, at the time of speciation, sister species will initially have nonoverlapping spatial distributions (i.e., allopatric or parapatric speciation) (Pigot et al. 2010).

We used the Gillespie algorithm to sample the waiting times between colonization, local extinction, and speciation events. Specifically, the waiting time to the next event is determined by randomly drawing a value from an exponential distribution with a mean equal to the sum of the rates of the three events. Which event occurs is then determined randomly according to the relative summed rates of each event. Although per‐population rates of colonization, local extinction, and speciation are equivalent across species, species will differ in their probability of undergoing these events, due to differences in range size (i.e., number of populations). In particular, per species rates of colonization, local extinction, and speciation will increase with species geographic range size, while rates of colonization will also depend on differences in the shape and placement of species geographic distributions which determine the chance of invading a yet unoccupied cell. Simulations were terminated after *T* time units or following the complete extinction of the clade. The simulation was programmed in C++ and the code is available at https://doi.org/10.6084/m9.figshare.5437126.v2.

### GEOGRAPHIC AND PHYLOGENETIC METRICS

At the end of the simulation, species range size was calculated as the number of cells occupied by each species. From the record of speciation and extinction events, we determined the age of each species as the time since its origination (i.e., the identity of the parent species is retained across speciation events). During each unit time interval we calculated evolutionary turnover as the number of species extinctions (i.e., all populations of a species become extinct) divided by the number of speciation events (Weir and Schluter 2007). This quantity is informative about (im)balance in speciation‐extinction dynamics across evolutionary history. We quantified changes in net diversification rates over time using the Δ*r* statistic (Pigot et al. 2010; Etienne and Rosindell 2012), which is defined as the difference between the net diversification (logarithm of the change in number of lineages having extant descendants) of the second and first half of the simulation. While shifts in diversification dynamics within each half are not accounted for by Δ*r*, this metric has many advantages that make it suitable for our purpose. For instance, by varying the parameters in our model, a wide range of species richness values would be expected and Δ*r* is robust to tree size differences. Moreover for those cases where simulations have the same crown age, Δ*r* provides a fair comparison across *K*_L_ because calculating diversification rates (in each half) will be done over the same time duration. We expect Δ*r* to be equal to zero for constant rates of diversification, while negative values indicate a slowdown whereas positive values suggest an increase in diversification toward the present. By keeping track of ancestor‐descendent relationships, we reconstructed a phylogenic tree for the extant species and recalculated Δ*r* for these lineages. We also measured tree asymmetry with a normalized version of Sackin's index which in contrast to other commonly use metrics allows direct comparison between trees of different sizes (Blum and Francois 2005). Sackin's index (*S*) can take both positive and negative values, with higher values indicate greater imbalance and a pure birth process generating trees with *S* = 0.

### EXPLORING DIVERSITY‐DEPENDENT DYNAMICS UNDER DIFFERENT BIOLOGICAL SCENARIOS

We conducted exploratory simulations to identify combinations of region sizes *A*, local ecological limits *K*_L_, rates of speciation λ, colonization γ, and local extinction μ that would ensure the system reached equilibrium over the duration of the simulation (*T* = 35 time units) and did not result in a computationally unmanageable number of species (maximum *K*_R_ = 384 000). We defined equilibrium as the state of the system when regional richness is (dynamically) constant over time; in evolutionary terms, equilibrium is reached when speciation equals extinction. We note that the total duration of the simulation *T* and limit to richness *K*_R_ specified in our simulations are arbitrary, and that the dynamics described by our model are in theory relevant across phylogenetic scales, from the global dynamics of large clades unfolding over hundreds of millions of years, to the dynamics of small clades taking place within a single region over only a few million years. Because a short (long) duration of the simulation *T* is equivalent to specifying rapid (slow) rates of speciation, colonization, and local extinction, we generally kept the duration of the simulation *T* fixed (Fig. 2).

We varied the parameter values used in our simulations to explore a wide range of biological scenarios. First, while keeping all other parameters fixed, we examined the effects of varying the local ecological limit to diversity (*K*_L_ = 4, 12, 36, 1500). Because the size of the region was fixed at *A* = 256, this also had the effect of varying the total regional limit to diversity (*K_R_* = 1024, 3072, 9216, 384 000). In the case of *K*_L_ = 1500, local diversity never approached this limit within the timeframe of the simulation, thus approximating an unbounded model of diversity in which the limit to regional diversity *K*_R_ is essentially infinite. Second, to explore the independent effects of both geographic and ecological‐niche space, we simultaneously varied the local ecological limit *K*_L_ and the area of the region *A*, while keeping the regional limit to richness *K*_R_ fixed. We explored a scenario where the local ecological limit is low and the region is large (*K*_L_ = 1, *A* = 4096), a scenario where the local ecological limit is high but the region is small (*K*_L_ = 256, *A* = 16), and a scenario with intermediate values of *K*_L_ and *A* (*K*_L_ = 16, *A* = 256). A scenario with *K*_L_ = 4096 and *A* = 1, representing an extremely high ecological limit in combination with the smallest regional area possible, was not explored. We did not study this scenario because species range size will be 1 and during a speciation event, the entire population of a species would be selected to undergo speciation, that is change of species identity. In other words, every speciation event implies the extinction of the parental species, making the local richness never higher than 1. Third, for these ecological and geographical settings we examined different combinations of speciation λ, colonization γ, and local extinction μ (See Table S1 for all parameter combinations explored).

Because colonization in our model is restricted to occur only between spatially adjacent cells (i.e., local dispersal), range expansions, and diversification may be locally inhibited by the boundary or the region or by the presence of locally saturated communities, even before the entire region is filled with populations. To examine how these local effects may influence the expected dynamics we repeated our analysis allowing species to colonize any available cell in the region (i.e., global dispersal) rather than only those adjacent to already occupied populations.

For each combination of parameters, we performed 100 replicate simulations. When *K*_L_ = 1500, clade diversity was extremely large, greatly increasing the computation time. For this scenario, we therefore conducted 50 replicate simulations.

## Results

### THE DYNAMICS OF DIVERSIFICATION AND RANGE SIZE EVOLUTION UNDER ECOLOGICAL LIMITS

**Figure 1:**
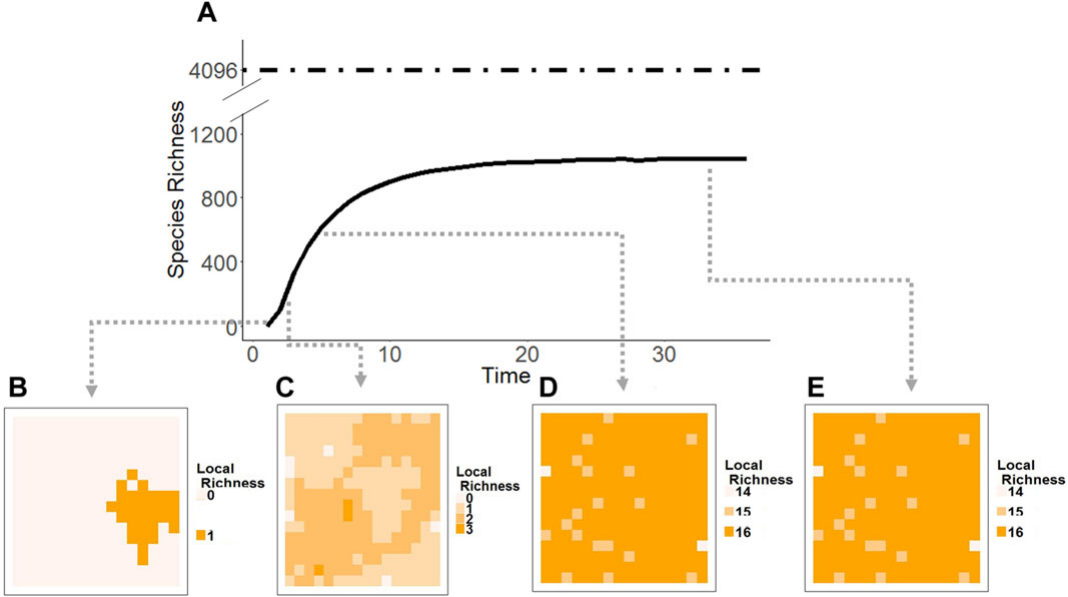
Changes in regional (A) and local (B–E) species richness over time under a model with a local ecological limit to diversity (*K_L_* = 16) in a bounded region (*A* = 256). Regional diversity initially increases rapidly but then asymptotes at a dynamic equilibrium (A). A single ancestral species expands its geographic range and produces new daughter species (B), leading to an increase in both local and regional richness (C). Local richness quickly saturates but ongoing allopatric speciation allows regional diversity to continue to increase but at a progressively slower rate (D). Finally, a steady state in regional species richness is attained (E). Local extinctions reduce local diversity and lead to the global extinction of species, resulting in a regional equilibrium that is lower than the theoretical maximum number of species (*K*_R_ = *K_L_* × *A*, dashed line) that can be packed within the region at saturation (i.e., each species comprises a single population). We used the following rates: λ = 0.08, γ = 80, μ = 1.

**Figure 2:**
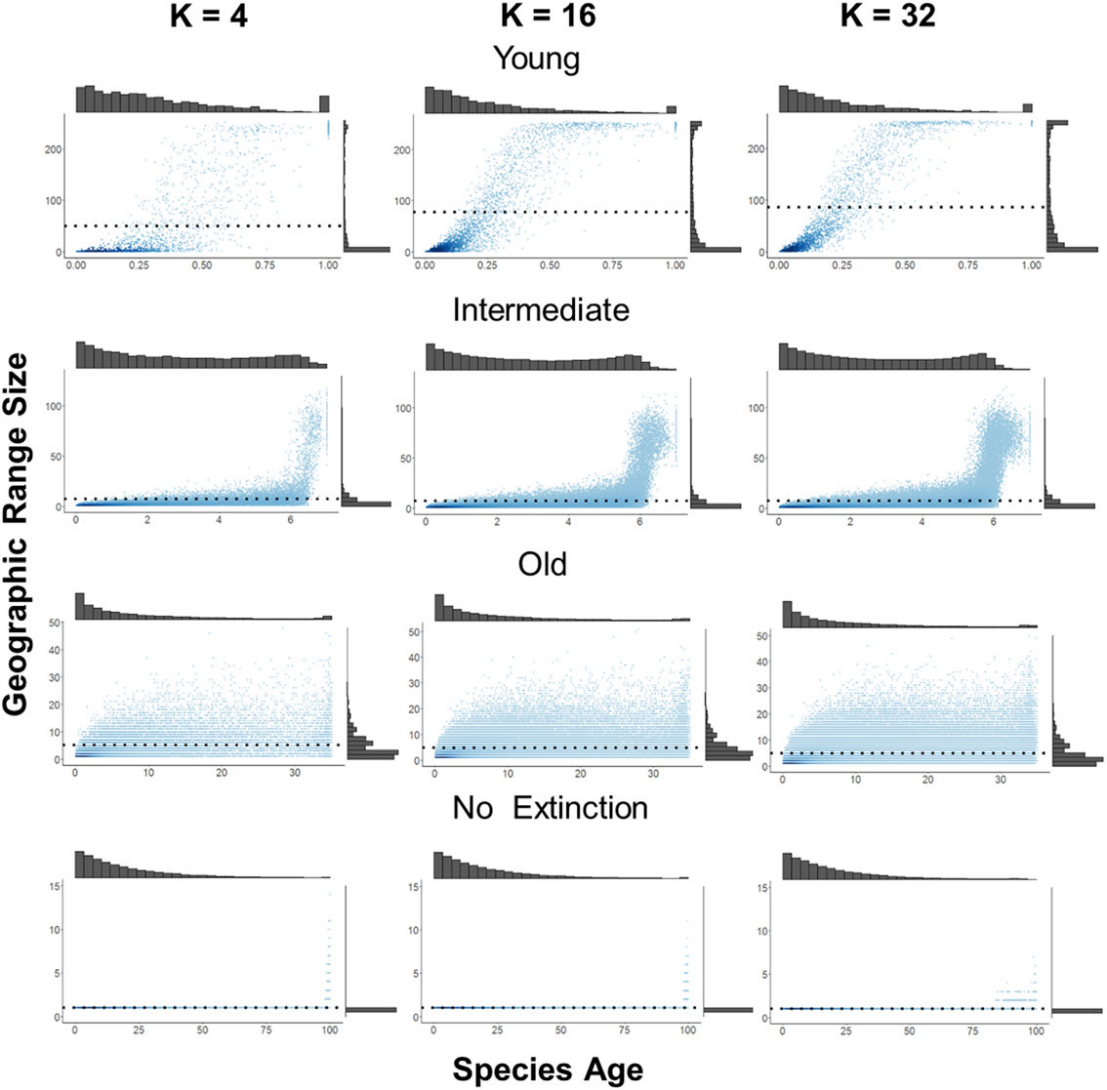
The evolutionary dynamics of geographic range size and its dependence on the local ecological limit to coexistence (*K_L_* = 4, 16, 32; columns) and clade age (i.e., the time since the beginning of the simulation; young = 1, intermediate = 7, old = 35). Points in each panel represent individual species from 100 replicate simulations, with darker colors indicating a higher density of points. Marginal histograms show the frequency distribution of species age (top) and range size (right). Dotted lines show the mean range size under each scenario. The area of the region (*A* = 256), the per population speciation rate (λ = 0.08), and colonization rate (γ = 80) were held constant across all scenarios while local extinction rate was either set to high (μ = 1, top three rows) or zero (μ = 0, bottom row). When local extinction (μ) = 0, the region becomes fully saturated with species so that each species has a range size of 1 cell at the time when saturation is achieved (*T* = 100).

**Figure 3:**
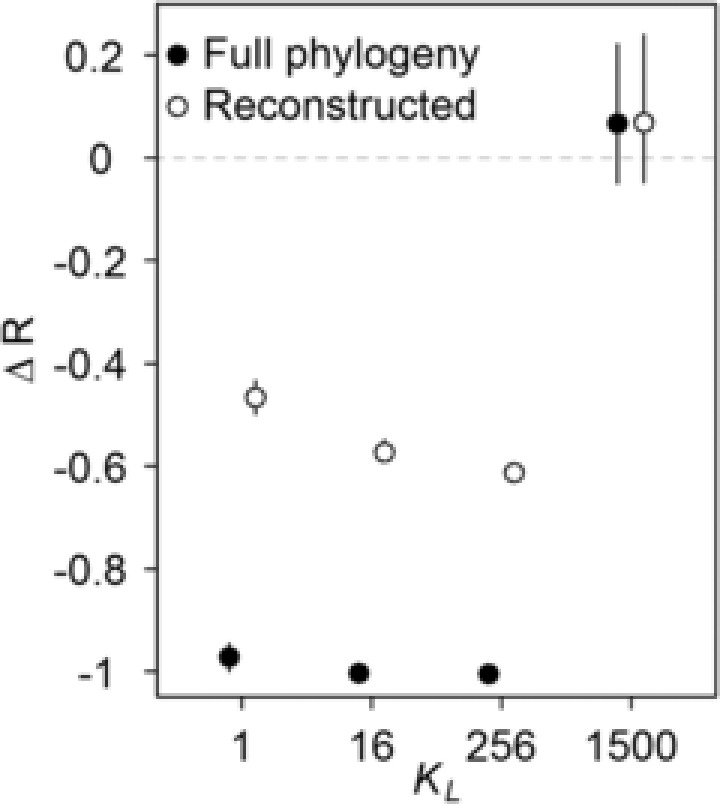
The effects of local (*K_L_*) ecological limits on the temporal dynamics of species diversification. The change in species diversification rate over time (Δ*r*) is shown for both the full phylogeny (including extinct species, solid symbol) and the reconstructed phylogeny (excluding extinct species, empty symbol) for different local ecological limits (*K_L_* = 1, 16, 256). Regional area is varied (*A* = 4096, 256, 16 cells) so that the regional limit to richness (*K*_R_) is kept fixed. In the case of *K_L_* = 1500 the area is 256. Δ*r* indicates the relative diversification rate in the first and second half of the simulation. When Δ*r* < 0, lineage accumulation slows down whereas when Δ*r* > 0, lineage accumulation speeds up. The mean (and 95% confidence interval) in expected Δ*r* is shown for 100 replicate simulations.

Starting from a single population, the ancestral species in our simulation expands its range through the process of colonization. Some of these populations become new species leading to an increase in regional richness (Fig. 1A). In these early stages of diversification, and so long as rates of colonization are faster than rates of speciation, average species range size tends to increase with species evolutionary age (Fig. 2, top panel). The increase in species’ range sizes in a young clade leads to an initially accelerating rate of speciation and declining rate of extinction, and thus an increasing rate of diversification over time. This transient increase in diversification rate is most evident under simulations where local ecological limits are extremely high (Fig. 3, *K*_L_ = 1500), and thus where species assemblages remain far from saturation over the duration of the simulation (final mean local richness across cells = 502, with the parameters of Fig. 3).

**Figure 4:**
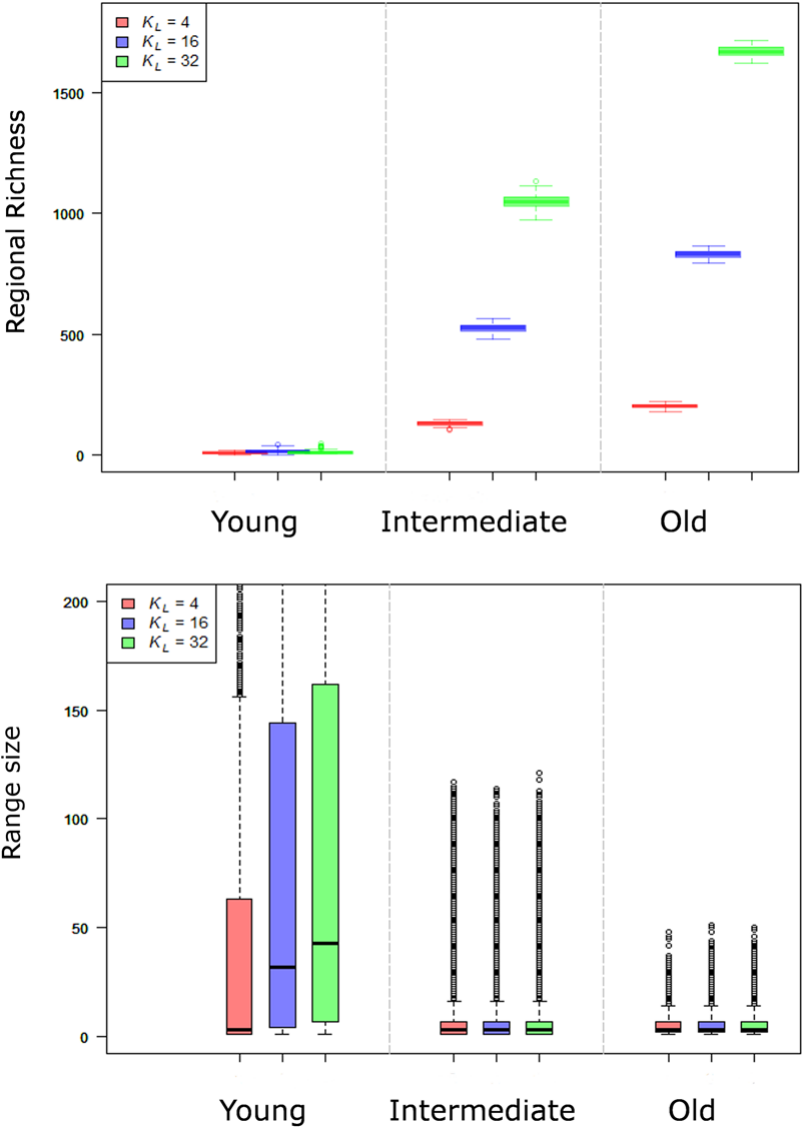
The effect of differences in local ecological limits (*K*_L_ = 4, 16, 32) on regional species richness and geographic range size and at different times in a clade's history (young = 1 my, intermediate = 7 my, old = 35 my). The per population speciation rate (λ = 0.08), rate of colonization (γ = 80), rate of local extinction (μ = 1), and regional area (*A* = 256) were the same across simulations. Results are based on 100 replicate simulations per scenario.

As species colonize assemblages local diversity increases (Fig. 1B–E). When there are ecological limits to local diversity *K*_L_, communities become saturated with species preventing further colonization events. Because colonization is hindered, newly formed species are unable to expand their distributions and thus range sizes remain small long after speciation (Fig. 2). In addition, the geographic ranges of widespread species start to contract because rates of per‐population speciation and local extinction now exceed rates of colonization (Fig. 2). Together, these processes lead to a decline in species mean range size (Fig. 4), which in turn leads to an increase in the per‐species rate of extinction and also a decline in the per‐species rate of speciation. As a result, rates of species diversification start to slow down (Fig. 3). Eventually, the clade reaches a dynamic equilibrium whereby rates of speciation are balanced by rates of extinction and diversity remains approximately constant over time (Fig. 1A). This equilibrium in regional diversity is also reflected in approximately constant levels of mean local richness and species geographic range size.

In the presence of ecological limits to local diversity a strong temporal slowdown in diversification rate is visible when using the reconstructed diversification process, but this is considerably weaker than the true diversification slowdown (Fig. 3). This is because, as previous simulation studies have shown (Quental and Marshall 2009; Liow et al. 2010), high or accelerating rates of species extinction erode the signature of slowdowns in phylogenies containing only extant lineages. Interestingly, for a constant limit to regional diversity *K*_R_, the strength of the slowdown in the reconstructed phylogeny depends on the relative values of *K*_L_ and regional area *A* (Figs. 3 and 4). In particular, when the limit to local richness *K*_L_ is reduced to a very low level (and thus *A* is large), evidence of a slowdown in the reconstructed rate of diversification becomes weaker (*K*_L_ = 1: Δ*r*_mean_ = –0.46) compared to a scenario with intermediate values of *K*_L_ and *A* (*K*_L_ = 256: Δ*r*_mean_ = –0.61) (Fig. 3). This is because low local limits to diversity *K*_L_ inhibit geographic range expansions, leading to faster rates of species extinction, thus eroding the signature of a temporal slowdown in diversification rate from the reconstructed tree. When Δ*r* is calculated using the full tree, the slowdown is similar in both scenarios (Δ*r*_mean_ = –0.971 for *K*_L_ = 1 and Δ*r*_mean_ = –1.004 for *K*_L_ = 256).

### PATTERNS OF REGIONAL SPECIES RICHNESS AT EQUILIBRIUM

**Figure 5:**
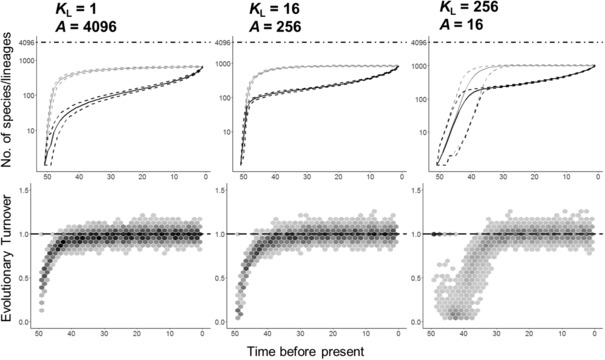
Temporal patterns in species richness, lineage accumulation, and evolutionary turnover (the ratio of extinction to speciation rate) for different local ecological limits (*K*_L_ = 1, 16, 256) in combination with different regional area (*A* = 4096, 256, 16 cells) thus keeping the regional limit to richness (*K*_R_) fixed. The top row shows the regional species richness (gray line) and the number of lineages in the reconstructed phylogeny over time (black line) along with their 95% confidence intervals (dashed lines) across 100 simulations. The horizontal line represents the maximum potential number of species the region can hold (*K*_R_ = *K_L_* × *A*). The bottom row shows the dynamics of speciation and extinction over time. When evolutionary turnover equals 1 (dashed lines) rates of speciation and extinction are identical. Values lower (greater) than 1 indicate when rates of speciation are greater (less) than extinction. Darker cells indicate a higher concentration of observations. In each case, speciation rate (λ) = 0.05, colonization rate (γ) = 30, and local extinction (μ) = 1.

**Figure 6:**
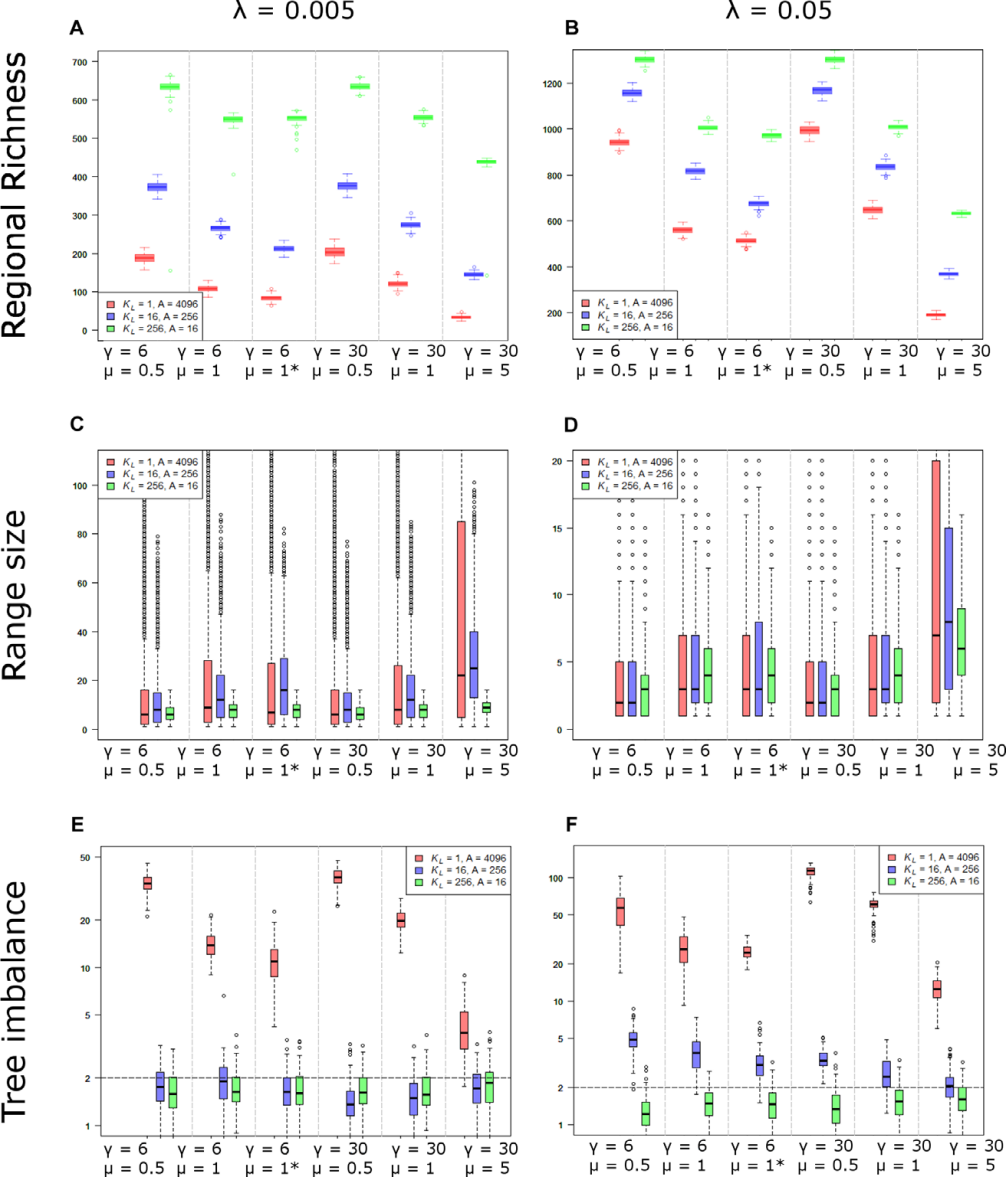
Regional richness, range size, and tree imbalance (Sackin index) under different diversification scenarios. We explored scenarios encompassing different combinations of local carrying capacity (*K*_L_) and regional area (*A*), rates of colonization (γ), local extinction (μ), per population speciation (λ), and mode of dispersal. For each combination of parameters, a local dispersal model is assumed, except in the cases marked with an “*” that assumes a global model of dispersal. The duration of the simulation (*T* = 35) and the potential regional diversity limit (*K*_R_ = 4096) was held constant across all scenarios. Results are based on 100 replicate simulations per scenario. N.B the scale of the *y*‐axes differs between plots. In (E, F), tree imbalance according to Sackin's index is plotted on a log‐scale with the dashed horizontal line indicating the expected level of tree imbalance under a Yule process (Sackin = 0). Because Sackin's index may take values < 0, we added a value of 2 to the index prior to log‐transformation, that is log(Sackin + 2).

The regional richness attained at equilibrium depends on *K*_L_, *A* and the relative rates of speciation, local extinction, and colonization (Figs. 4 and 6). As expected larger values of *K*_L_, and thus regional diversity limit *K*_R_, lead to a higher regional richness at equilibrium (Fig. 4). This occurs because a higher *K*_R_ supports more species populations and thus, for a given level of richness, faster rates of speciation and slower rates of species extinction. For a given *K*_R,_ lower per‐population rates of speciation λ lead to a lower equilibrium regional richness (Fig. 6A) compared to when per‐population rates of speciation λ are high (Fig. 6B). Conversely, higher rates of local extinction μ lead to a lower equilibrium richness because of an increase in the rate of species extinction (Fig. 6). Indeed, in the absence of local (and thus species) extinction (μ = 0), clades would eventually saturate the region, whereby every assemblage contains *K*_L_ species and each species is present in only a single local assemblage, that is all species have a range size of 1 (Fig. 2, bottom panel). In contrast, when rates of local extinction μ exceed zero, regional diversity is maintained at a dynamic equilibrium lower than the theoretical upper limit to diversity (Figs. 1 and 5). Unless rates of colonization γ are very low, variation in γ has relatively little effect on regional richness (Fig. 6A and B). This is because, at equilibrium, rates of colonization are limited by the rate at which local sites become available following local extinction rather than by the colonization rate γ.

In the absence of extinction, the region eventually becomes saturated with species (i.e., *K*_R_ species) and this upper limit to diversity is the same regardless of the relative values of *A* and *K*_L_ (*K*_R_ = *K*_L_ × *A*)_._ However, when rates of local extinction are greater than zero, we found that geographic area *A* and local diversity limits *K*_L_ have different effects on the accumulation of diversity and the regional richness attained at equilibrium. Specifically, it takes longer to reach equilibrium (Fig. 4) but diversity attains a higher level (Fig. 6A and B) when the geographic area of the region *A* is small but the local ecological limit *K*_L_ is high, than when the area of the region *A* is large but the local ecological limit *K*_L_ is small. This difference in richness is robust to differences in rates of speciation λ and colonization γ and strengthens with higher rates of local extinction μ (Fig. 6). For instance, when the rate of local extinction is low (μ = 0.5), a small region with a high local ecological limit (*A* = 16, *K*_L_ = 256) has a regional richness that is 1.3 times that of a large region with a low local ecological limit (*A* = 4096, *K*_L_ = 1), but when rates of local extinction are high (μ = 5) the difference in regional richness increases to fivefold (Fig. 6B).

### PATTERNS OF PHYLOGENETIC TREE IMBALANCE

We found that clades diversifying in regions with extremely high local ecological limits (*K*_L_ = 1500, *A* = 256), and which are far from reaching local saturation, exhibit a similar level of phylogenetic tree imbalance to that expected under a Yule process (*S*_mean_ = –0.08). In contrast, when local ecological limits are lower (i.e., impose greater constraints on diversification), the expected shape of phylogenetic trees varies greatly depending on the relative size of the geographic region *A* and local ecological niche space *K*_L_, rates of speciation λ, local extinction μ, and colonization γ (Fig. 6E and F). In particular, when the region is large but local ecological limits are low (*A* = 4096, *K*_L_ = 1), phylogenetic trees are highly unbalanced. This arises because newly formed species have small geographic ranges leading to large asymmetries in range size and thus probabilities of speciation and extinction (Fig. 2). By contrast, in small regions with high local ecological limits, phylogenetic trees are more balanced than expected under a pure birth process (*A* = 16, *K*_L_ = 256). This arises because a small geographic area constrains species to have relatively small geographic ranges (Fig. 6C and D). Species undergoing rapid speciation will thus exhibit large proportional declines in range size, thus substantially decreasing the probability of further speciation and increasing the chances of extinction. This negative feedback on the diversification of rapidly speciating lineages leads to more balanced phylogenetic trees. Higher rates of local extinction μ and colonization γ, relative to rates of speciation λ, lead to phylogenetic trees converging on the shape expected under a pure birth process (Fig. 6E and F). This is because rapid extinction‐colonization dynamics erode the signature of past speciation history on geographic range size, thus equalizing probabilities of diversification across lineages.

### THE PATTERNS AND TEMPORAL DYNAMICS OF GEOGRAPHIC RANGE SIZE

For a given regional area *A*, and when the local ecological limit *K*_L_ is low, communities rapidly become saturated with species thus inhibiting geographic range expansions. As a result, species retain a small geographic range for longer periods of time following speciation. In contrast, when constraints on local richness are relaxed (i.e., the local ecological limit *K*_L_ is higher), there is more time available for range expansion before local communities become saturated leading to larger mean range sizes and a stronger relationship between species age and range size (Fig. 2). This effect of *K*_L_ on species range size is, however, transient. When richness reaches a regional equilibrium, mean geographic range size becomes independent of the local *K*_L_ and thus regional *K*_R_ limit to richness (Figure 4). This is because mean range size is maintained at a dynamic equilibrium set by a balance between rates of local extinction μ and speciation λ, which act to reduce range size, and the rate of colonization γ, which acts to increase range size. Furthermore, for a given limit to regional diversity *K*_R_, although clades occupying smaller regions (i.e., *A* is low) exhibit less variation in range size than clades occupying large regions (i.e., *A* is high), there is relatively little variation in mean range size (Fig. 6C and D).

Although local extinction events reduce species’ range size, we found that mean species range size actually increases with the rate of local extinction μ (Fig. 6C and D). We argue that this happens via two coupled mechanisms: first, when rates of local extinction μ are high, species with small geographic ranges are more likely to become extinct. Second, the ecological space left by extinct species becomes available to be colonized by large‐ranged species that implies further range expansion. Therefore both the selective filtering of rare species and the expansion of widespread species lead to an increase in mean range size among the survivors. As expected, a higher speciation rate λ leads to smaller range sizes, but the rate of colonization γ had surprisingly little effect on range size (Fig. 6C and D). Indeed, our simulations also showed that the characteristics of clades at equilibrium are rather insensitive to whether dispersal is local or global (Fig. 5). This is because once communities are saturated with species, range expansions become limited by the rate at which local extinctions open up opportunities for colonization rather than the intrinsic rate of colonization per se. Thus, at equilibrium, higher dispersal capacities either in the form of global dispersal or higher colonization rate play a secondary role in range expansions and the accumulation of diversity.

The shape of the range size frequency distribution at equilibrium is strongly right skewed, with many small‐ranged species and only a few widespread species (Fig. 2). This pattern is generally evident regardless of the values of *K*_L_, λ, and γ. In contrast, in the absence of local extinction (μ = 0), ongoing speciation leads to a continuous decline in range size so that all species eventually have a range size of 1. While a right skewed distribution with a single mode predominates at equilibrium, a bimodal distribution in range size may emerge early in the clade's history (Fig. 2). This is because early arising species rapidly expand their distributions throughout the region but this subsequently inhibits the expansion of later arising species. This bimodal pattern is, however, a transient phenomenon and as diversity reaches a steady state, the range size distribution shifts to becoming increasingly right‐skewed with a single mode. This smoothing of the range size distribution occurs because speciation and local extinction gradually erode the range size of the most widespread species, while rare species gradually expand their distributions as they invade spaces in local communities left empty by local extinction.

## Discussion

With a spatially explicit model of species diversification we explored the phylogenetic and geographic patterns expected when regional limits to diversity arise from local limits to coexistence. Most mathematical models of diversity‐dependent diversification (Rabosky and Lovette 2008; Etienne et al. 2012) assume a direct connection between clade richness and rates of speciation and extinction. In contrast, in our model diversity‐dependence in these macroevolutionary parameters arises as an emergent property of local limits to coexistence.

Our model shows that when local diversity is bounded, rates of species diversification decline over time. This occurs because as local assemblages become ecologically saturated, newly formed species are unable to expand their distributions thus preventing further rounds of speciation and increasing rates of extinction. Eventually regional species diversity reaches a steady state in which the number of species and their average geographic range size fluctuate around an equilibrium set by the relative rates of speciation and extinction.

Previous studies, using nonneutral models, have also shown how diversity‐dependence in rates of diversification can emerge from local processes. For instance, in models of adaptation radiation (Gavrilets and Vose 2005; Birand et al. 2012), an initially high rate of diversification precedes a decrease in the rate of speciation and an increase in the rate of extinction. Pontarp and Wiens (2017) modeled species’ trait distributions as a function of local resources, the utilization of which determines the local species carrying capacity. In these nonneutral models high ecological niche availability initially promotes high rates of ecological speciation and thus an early burst in species diversification. Here, we modeled ecological opportunity in a simpler way (simply as the number of locally available niches), but our model nevertheless provides a number of novel insights into how geographic and local ecological niche diversity regulates species radiations.

One intriguing result from our model relates to how the relative dimensions of geographic and ecological‐niche space influence biodiversity. We found that small regions with high local ecological limits are able to support more species at equilibrium than large regions with low ecological limits, especially when rates of stochastic population extinction are high. This result may appear surprising because in our model the theoretical upper limit to diversity–occurring when each species comprises only a single population–is the same regardless of the relative dimensions of geographic and ecological‐niche space. The most likely explanation for this finding is that a high local ecological limit within a small geographic area maximizes the number of species that can regionally coexist by favoring the geographic spread, and thus persistence, of rare relative to widespread species. This arises because the area of the region ultimately constrains the maximum number of populations that a species can attain, thus preventing any single species from monopolizing the entire regional niche space. In other words, when an opportunity for colonization becomes available through the local extinction of a resident species, this opportunity can only be exploited by species absent from the local community, which by definition will tend to be relatively rare. In contrast, in a region where each locality contains only a single niche (*K*_L_ = 1), species that are already widespread are more likely to be available to exploit gaps left by recent extinctions, so that rare species may rapidly drift to extinction. This finding has a number of important implications. First, it suggests that differences in the diversity of regions and clades may be more strongly driven by differences in local ecological niche space than regional area. Second, variation in diversity between clades or regions may to a certain extent be uncoupled from variation in overall diversity limits.

In addition to the effects on overall regional diversity, we found that the relative size of geographic and local ecological niche space also influenced the structure of diversity within regions. In particular, we found that when local ecological limits are low (low *K*_L_), diversity is distributed highly asymmetrically across clades (i.e., phylogenetic trees are unbalanced). In a similar scenario (low number of available niches mediated via competition). Gascuel et al. (2015) found that phylogenetic trees show high imbalance and claimed that this is a trace of allopatric speciation taking place early in the history of a clade. In our model, the imbalance arises because newly formed species tend to be rare leading to highly skewed range size distributions and thus asymmetries in rates of speciation and extinction. In contrast, when local niche diversity limits are high (high *K*_L_) and geographic limits are small (small *A*), range expansion among widespread species is constrained relative to that of newly formed species, leading to more balanced phylogenetic trees. These results make the novel prediction that patterns of phylogenetic tree shape should vary predictably with local ecological niche diversity.

While our model shows that local limits to diversity must eventually lead to a steady state in regional richness, these local and regional dynamics may be partially decoupled. In particular, even when all local communities are saturated with species, ongoing allopatric speciation and the increasing turnover of species between assemblages can enable regional diversity to increase for long periods of time before the system reaches a dynamic equilibrium (Cornell 2013). An important implication of this result is that even if there is evidence that local diversity is saturated, this need not imply that the diversity of the entire region has reached a carrying capacity. Equally, even when total clade diversity is still increasing, this should not be taken as evidence that there are no ecological limits to local diversity or that any such limits have yet to be reached.

One of the more surprising results revealed by our analysis, is that although imposing a limit to local species richness acts as a constraint on range expansion, the average range size attained by extant species is similar regardless of whether the local ecological limit is high or low. This is counterintuitive because species should more readily expand their distributions when local diversity is less constrained. We found that early in the radiation and when communities are far from saturated, this is indeed the case, with higher ecological limits (high *K*_L_) enabling species to attain large range sizes. However, rapid range expansion also leads to faster rates of speciation and thus the accumulation of species with small range sizes. These two processes balance one another so that mean range size is independent of the local ecological limit. Eventually, range sizes reach a dynamic equilibrium whereby increases in range size due to colonization are balanced by decreases in range size due to speciation and local extinction.

What determines the distribution of species range size has long been the subject of debate; explanations are sought in differences among species in niche breadth, environmental tolerances, or dispersal capacity (Tomasovich et al. 2015; Fenberg and Rivadeneira 2017). The right‐skewed distribution of range sizes observed in empirical datasets (Hecnar 1999; De Troch et al. 2001; Pither 2003; Reed 2003) was recovered in our model regardless of the strength of the local ecological limit or the other key macroevolutionary parameters (e.g., rates of speciation). This consistency in the shape of the range size frequency distribution suggests that this distribution contains little information about the underlying process structuring species distributions. There are a few studies reporting a bimodal range size distribution (Gaston et al. 1998; Mora and Robertson 2005; Scott et al. 2012). Scott et al. (2012) suggest that this bimodal distribution is the result of differential dispersal capacities among species. Our findings show that such a distribution can also arise early in a clade's history for ecologically identical species with local limits to diversity. Under this scenario, species arising early in the clade's history are able to spread throughout the entire region, attaining large range sizes. In contrast, species that are formed later (when local communities are saturated) can only expand their ranges when resident species become locally extinct, leading to slow rates of range expansion and a bimodal distribution in range size. However, our model also shows that this bimodal distribution is a transient phenomenon. Over time, speciation and local extinction events lead to a decrease in the range size of the most widespread species, so that the range size distributions becomes increasingly right‐skewed range with a single mode.

Conceptually, our model is strongly rooted in the existing birth‐death framework of macroevolution. First, as in existing birth‐death models, we assume that species (or more precisely in our case species populations) are neutral in the sense that they are governed by identical dynamics. Second, as in existing diversity‐dependent birth‐death models, we assume that there is a limit to the number of species that a clade can support, but in our case this limit arises from a combination of both local ecological and spatial constraints. On the one hand, the simplicity of this extension to the existing birth‐death framework represents a key strength of our model, because it allows us to identify how simply considering space influences the expected dynamics of diversification. On the other hand, the way we have modeled ecological and geographical limits to richness still represent a major abstraction of reality. We therefore see room for much further refinement and extension of our model. For instance, limits to coexistence could be modeled as a result of individual level dynamics rather than at the level of species (Chesson 2000; Hillerislambers et al. 2012). Another obvious extension would be to incorporate a model of trait evolution, so that traits determine the place of a species within niche space. In this case, ecological constraints on coexistence could be modeled by making colonization/extinction a positive/negative function of ecological dissimilarity (Jiang et al. 2010; Pigot and Etienne 2015). Finally, the interplay between range evolution and diversification might be affected by the mode of speciation. Our model assumes that new species are initially rare, comprised of a single local population. However, other models of speciation, including vicariance driven by geographic barriers, may enable new species to inherit a large range size from their parent. This could produce different patterns of diversification and range size evolution. For instance, more symmetrical splitting of species ranges during speciation is expected to lead to more balanced phylogenetic trees (Pigot et al. 2010), while the drastic reduction in range size accompanying each speciation event would potentially leading to a faster attainment of a regional diversity equilibrium.

## Conclusions

Here, we developed a spatially explicit model of diversity‐dependent diversification to explore the phylogenetic and biogeographic patterns expected when ecological limits to diversity arise from local‐scale species interactions. While our findings demonstrate how local ecological limits lead to a predictable dynamic equilibrium in regional diversity and range size, they also highlight that variation in regional richness can to a certain extent be decoupled from variation in regional diversity limits, depending on the relative size of geographic and local ecological niche space and the relative rates of speciation, extinction, and colonization. Taken together, these findings provide an important bridge for understanding how large‐ and small‐ scale processes interact and further unite evolutionary and ecological mechanisms for understanding patterns of species diversity.

## CONFLICT OF INTERESTS

The authors have declared no conflict of interest.

Associate Editor: F. Debarre

Handling Editor: Mohamed A. F. Noor

## Supporting information


**Table S1**. Parameter values explored in the simulations.
