# Supplementary material for: The influence of ecological and geographic limits on the evolution of species distributions and diversity
Source: Evolution. 2018 Aug 7;72(10):1978–91. doi: 10.1111/evo.13563 (PMC6220796; doi:10.1111/evo.13563)
Supplement: Supplementary file 1 — Table S1. Parameter values explored in the simulations. [file EVO-72-1978-s001.docx]

Table S1. Parameter values explored in the simulations.

| Speciation (λ) | Colonization (γ) | Local extinction (µ) | Area | *K*_L_ |
| --- | --- | --- | --- | --- |
| 0.08 | 80 | 0 | 256 | 4,16,36 |
| 0.08 | 80 | 1 | 256 | 4,16,36 |
| 0.05 | 30 | 5 | 4096, 256, 16 | 1, 16, 256 |
| 0.05 | 30 | 1 | 4096, 256, 16 | 1, 16, 256 |
| 0.05 | 30 | 0.5 | 4096, 256, 16 | 1, 16, 256 |
| 0.05 | 6 | 1 | 4096, 256, 16 | 1, 16, 256 |
| 0.05 | 6 | 1 | 4096, 256, 16 | 1, 16, 256 |
| 0.05 | 6 | 0.5 | 4096, 256, 16 | 1, 16, 256 |
| 0.005 | 30 | 5 | 4096, 256, 16 | 1, 16, 256 |
| 0.005 | 30 | 1 | 4096, 256, 16 | 1, 16, 256 |
| 0.005 | 30 | 0.5 | 4096, 256, 16 | 1, 16, 256 |
| 0.005 | 6 | 1 | 4096, 256, 16 | 1, 16, 256 |
| 0.005 | 6 | 1 | 4096, 256, 16 | 1, 16, 256 |
| 0.005 | 6 | 0.5 | 4096, 256, 16 | 1, 16, 256 |
